# Five-Year Evaluation of a Multilingual Website on Pregnancy and Parenting for Foreign Families in Japan

**DOI:** 10.7759/cureus.94592

**Published:** 2025-10-14

**Authors:** Masumi Moriyama, Mami Gomi, Yukari Kamei, Eri Mochida, Haitang Xie, Aya Nitamizu, Yae Yoshino, Mikako Arakida

**Affiliations:** 1 Nursing, Japanese Red Cross Kyushu International College of Nursing, Munakata, JPN; 2 Nursing, Kawasaki City College of Nursing, Kawasaki, JPN; 3 Nursing, Shitennoji University, Habikino, JPN; 4 Nursing, Oita University of Nursing and Health Sciences, Oita, JPN; 5 Nursing, Graduate School of Nursing, Seitoku University, Matsudo, JPN; 6 Nursing, Tokyo Medical University, Tokyo, JPN; 7 Nursing, Faculty of Human Sciences, Sophia University, Tokyo, JPN

**Keywords:** digital health platform, easy japanese, foreign-origin families in japan, maternal and child health, multilingual health communication

## Abstract

Background: Japan’s increasing population of foreign residents includes many of reproductive age who face barriers in accessing maternal and child healthcare, especially due to language and cultural differences.

Objective: This study aimed to evaluate the usage patterns, user satisfaction, and stakeholder engagement with a multilingual website (https://ecdsuishin.com) offering maternal and child health information to foreign-origin families in Japan.

Methods: A descriptive observational study was conducted between April 2020 and March 2025. Website access data were analyzed using Google Analytics (Google LLC, Mountain View, USA). User satisfaction was assessed through a multilingual, anonymous web-based survey. Inquiries submitted via the website were qualitatively analyzed. The Strengthening the Reporting of Observational Studies in Epidemiology (STROBE) checklist was used to guide study reporting.

Results: The website recorded 164,822 page views. The most frequently accessed languages were Japanese (n = 96,397, 59%), English (n = 24,872, 15%), and Easy Japanese (n = 9,658, 6%). Access was logged from 757 municipalities across all 47 prefectures. Survey respondents (n = 39) reported high satisfaction with usability. A total of 33 inquiries highlighted diverse needs for multilingual support and professional assistance.

Discussion: The findings support the role of multilingual digital platforms in improving health equity. Culturally and linguistically appropriate content supports both families and healthcare providers by reducing access barriers.

Conclusion: Multilingual, culturally responsive digital platforms play a crucial role in reducing barriers to maternal and child health information among foreign-origin families in Japan. Furthermore, to advance health equity and ensure inclusive access to healthcare, it will be necessary to expand the content of these platforms, conduct targeted outreach, and integrate them into broader health systems.

## Introduction

Current state of foreign residents in Japan and challenges in maternal and child healthcare

In recent years, the number of foreign residents in Japan has steadily increased, driven by globalization and changes in labor market structures. As of 2024, the foreign resident population exceeds 3.5 million [1], with a large percentage being of reproductive and child-rearing age [2]. According to national vital statistics, approximately 20,000 foreign women give birth in Japan annually [3].

Although various support systems have been developed by governmental and non-governmental organizations, many foreign families still face substantial barriers in accessing maternal and child health services. Persistent challenges include language barriers and the lack of accessible multilingual health information [4,5]. We previously reported that in municipalities with large foreign populations, only 60-70% of infant health checkups utilized translated questionnaires or public interpreters [6].

These barriers not only hinder access to health-related information but also have direct and significant implications for the health of foreign mothers and their children. Our previous web-based survey targeting foreign mothers revealed that some participants were unaware of essential maternal and child health services, such as the Maternal and Child Health Handbook, prenatal checkups, parenting classes, vaccinations, and infant health screenings, and thus had not accessed them accordingly [7]. Other studies identified similar issues, such as delayed vaccinations, missed health checkups, postpartum isolation, and deteriorated mental health conditions. Moreover, foreign women often experience confusion, anxiety, and feelings of isolation due to unfamiliarity with the Japanese healthcare system, cultural gaps, and a lack of community-based support. These experiences have been associated with increased risks of high-risk pregnancies and postpartum depression [8,9].

Need for multilingual digital platforms

While some healthcare institutions offer partial language assistance, most existing services fall short of addressing the complex needs of culturally and linguistically diverse populations. Specifically, volunteer interpreting services have limitations in providing continuous support, and information provision is often biased toward certain languages, failing to cover the diverse linguistic backgrounds of all users. The Japanese government is currently promoting the digitalization of maternal and child health information and the utilization of related data. However, the current provision of multilingual digital platforms that are comprehensive, culturally appropriate, and easy to understand, particularly those specialized in maternal and child health, remains insufficient [10]. Evidence suggests that multilingual e-health platforms improve access to information, promote informed decision-making, and empower users from culturally and linguistically diverse backgrounds [11,12]. However, there is limited systematic research in Japan that comprehensively examines the long-term usage patterns of multilingual digital platforms specifically in the maternal and child health sector, or their concrete impact on user satisfaction and behavior.

Purpose of this study

The purpose of this study was to evaluate usage patterns, user satisfaction, and stakeholder engagement with a multilingual maternal and child health website (https://ecdsuishin.com) in Japan in order to generate evidence that can inform inclusive health communication policies and support the development of a sustainable model for providing equitable health information to foreign residents. By aligning these objectives, the study seeks to offer practical insights that can guide future improvements in maternal and child health services and inform the application of multilingual information provision in other healthcare fields.

## Materials and methods

Study design and period

A descriptive longitudinal study was conducted to evaluate website usage trends and user satisfaction over a five-year period (April 2020-March 2025).

Website usage data were continuously collected through the website’s analytics system, summarized monthly, aggregated annually, and then compiled across the five-year study period. User satisfaction feedback was reviewed as it was submitted, summarized annually, and compiled across the same five-year period. Because these two data sources differed in nature, with one consisting of automated analytics data and the other consisting of user-provided feedback, they were analyzed separately, and the results were compared to identify patterns in website utilization and perceived usefulness.

This study is reported in accordance with the Strengthening the Reporting of Observational Studies in Epidemiology (STROBE) checklist for observational studies [13].

Website description

With the increasing number of foreign residents in Japan, the population of children from diverse cultural and linguistic backgrounds has also grown. Although multilingual materials on maternal and child health and parenting support have been developed, their dissemination and utilization remain insufficient.

To address these challenges, our research team launched a project to create a database on early childhood development (ECD) in both the countries of origin of foreign residents and in Japan. As part of this initiative, we conducted a Delphi study with public health nurses involved in maternal and child health programs, reviewed relevant literature, and systematically collected and assessed the accuracy of information available on maternal and child health websites.

**Figure 1:**
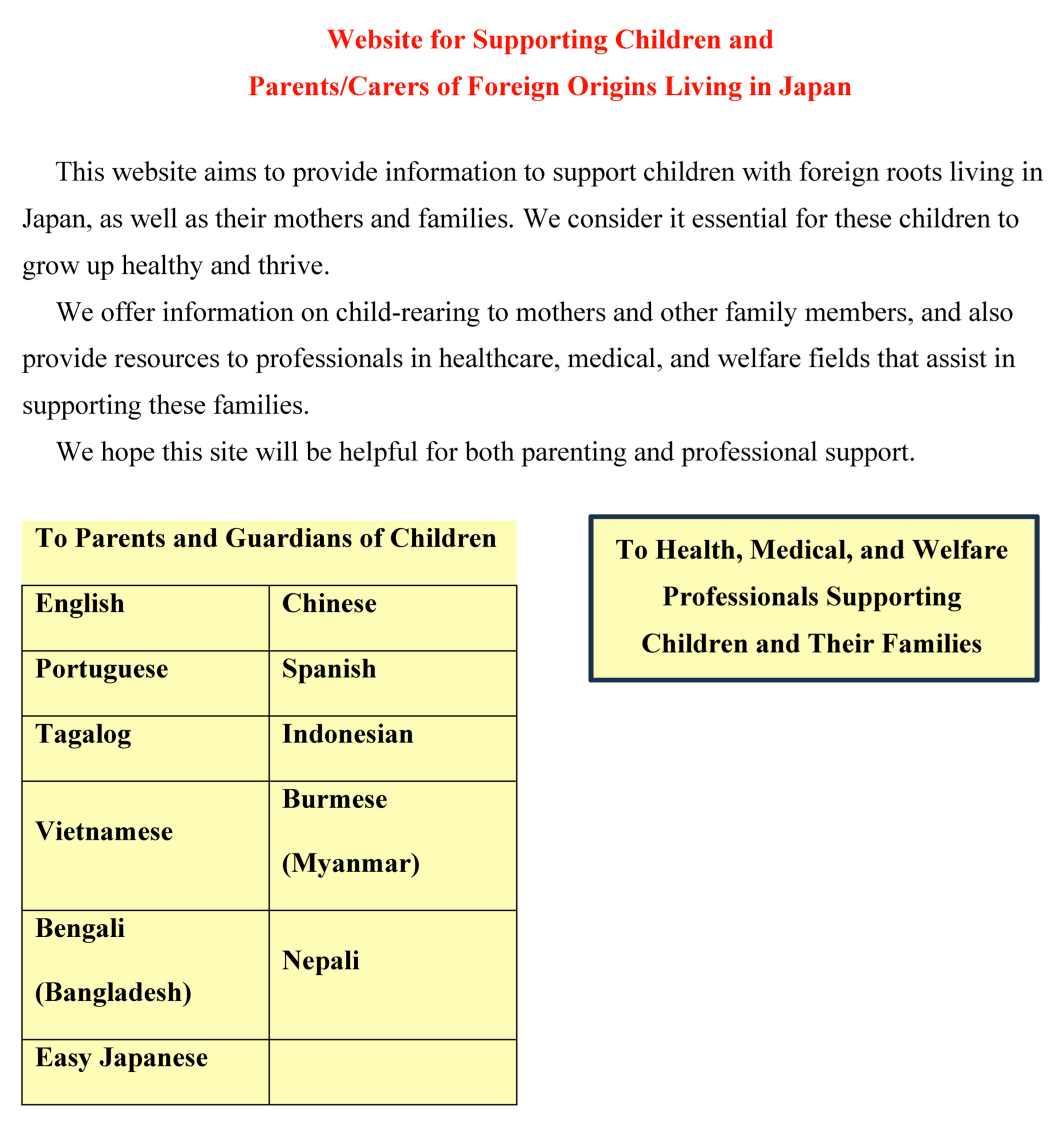
Screenshot of the homepage of the multilingual information website for supporting children and parents/carers of foreign origin in Japan Source: Translated English version of the original Japanese homepage from https://ecdsuishin.com, a website by the Research Association for Promoting Early Childhood Development for Children With Foreign Roots Living in Japan.

Building on these efforts and research findings, we subsequently launched a multilingual information website for pregnancy, childbirth, and childcare support for foreign residents (https://ecdsuishin.com) in 2020 to provide foreign-origin families in Japan with reliable and accessible maternal and child health information (Figure 1).

The website was designed to serve two primary user groups: (i) foreign residents who are parents or guardians of children, and (ii) healthcare, social welfare, and public health professionals who work with culturally and linguistically diverse populations. By offering content tailored to each audience, the platform enhances both personal and professional understanding and communication in perinatal and pediatric health settings.

The website offers information in 11 languages, including English, Chinese, Portuguese, Spanish, Tagalog, Vietnamese, Indonesian, Burmese, Bengali, Nepali, and Easy Japanese, thereby enhancing accessibility for diverse users. The selection of these languages was based on the demographics of foreign residents in Japan at the end of 2019 [14]. At that time, the largest groups by nationality were Chinese, followed by Koreans, Vietnamese, Filipinos, Brazilians, Nepalis, Indonesians, Taiwanese, Americans, Thais, Peruvians, Indians, Myanmarese, Sri Lankans, Mongolians, French, Russians, British, Canadians, Germans, and Bangladeshis. Notably, many Koreans had already been residing in Japan for several generations, from the first through the fourth. Based on these demographic data, we selected Chinese, Vietnamese, Tagalog (Philippines), Portuguese (Brazil), Nepali, Indonesian, Burmese, Bengali (Bangladesh), Spanish (Peru and other Latin American residents), and English (United States, United Kingdom, Canada, etc.), in addition to Easy Japanese, resulting in a total of 11 languages. The inclusion of these languages was further facilitated by the availability of Japanese researchers and foreign residents in Japan who could provide both linguistic and cultural support.

Its content covers a wide range of maternal and child health topics, including the use of the Maternal and Child Health Handbook, prenatal care, preparation for childbirth, postpartum checkups, vaccination schedules, child development, and navigating the healthcare system in Japan. In addition, the website provides culturally informed resources, such as downloadable multilingual materials, visual communication tools (e.g., pointing boards for maternity visits), and guides that explain healthcare systems and cultural practices in users’ countries of origin. Dedicated pages for healthcare and welfare professionals offer links to local government websites in multicultural regions, printable multilingual flyers, and culturally responsive educational materials. To further facilitate communication, a multilingual inquiry system allows users to submit questions in any of the 11 languages, including Japanese. Finally, the website is designed with a mobile-friendly interface to ensure convenient access across a variety of living and working environments.

By integrating research-based knowledge with practical information, the website functions as a unique and scalable model of digital multilingual support.

Data collection and analysis

Website analytics data were collected using Google Analytics (Google LLC, Mountain View, United States) to evaluate user behavior. The analysis covered a five-year period from April 2020 to March 2025.

The data were categorized and analyzed along three primary dimensions. First, language-specific access trends were examined by tracking page views across the 11 languages. Second, the website content was organized into thematic categories such as prenatal care, maternal health, child development, and immunization to identify the topics that generated the highest levels of user engagement. Finally, access patterns were analyzed based on users’ regional locations within Japan and compared with the prefecture-level distribution of foreign residents using the official statistics on foreign residents available through e-Stat, the Government of Japan’s official statistics portal [15].

Monthly page view data were downloaded and compiled using Microsoft Excel (Microsoft Corp., Redmond, United States). Customized dashboards were developed to visualize trends over time, compare usage across languages and content types, and highlight seasonal or regional variations in access. Descriptive statistics were used to summarize overall usage, and patterns were interpreted in relation to demographic data. Prefecture-level counts of foreign residents from e-Stat were used as a reference to provide context.

To assess user satisfaction, the authors conducted an anonymous web-based survey using a custom-developed evaluation scale, informed by the concept of the customer satisfaction score [16]. A dedicated section titled "Website Evaluation" was added to the platform, allowing users to access and complete the survey by clicking the corresponding link. The survey was available in multiple languages to accommodate the linguistic diversity of users. Respondents were asked to evaluate the following three domains using a four-point Likert scale: text clarity, content relevance, and usability. The collected data were analyzed using descriptive statistics to identify trends in satisfaction by language group and content category. The open-ended section invited users to provide suggestions for improvement regarding text readability, written content, page structure, or other aspects. In addition, a qualitative content analysis of these open-ended responses was conducted, following Krippendorff’s framework for content analysis [17], to identify common themes related to user experiences, positively perceived aspects, and recommendations for improvement. The number of inquiries was aggregated by fiscal year, and their content was qualitatively analyzed using the approach described by Krippendorff [17] to identify the types of information users were seeking and to provide additional context for interpreting user needs.

Ethical considerations

The present study utilized anonymized website usage data collected through Google Analytics. Users were not explicitly informed that these data might later be aggregated and analyzed for research purposes; this limitation is acknowledged here. Because the data were anonymized and originally collected for service improvement, institutional ethics approval was not required. Prior to the public release of the website’s content, informed consent was obtained from all collaborating organizations.

## Results

Website access metrics

Over the five-year evaluation period, the multilingual website garnered a total of 164,822 page views, with an average of 2,747 views per month. Notable spikes in access were observed during a promotional campaign in August 2020 (5,632 views) and following a seminar presentation in November 2020 (14,963 views).

**Figure 2:**
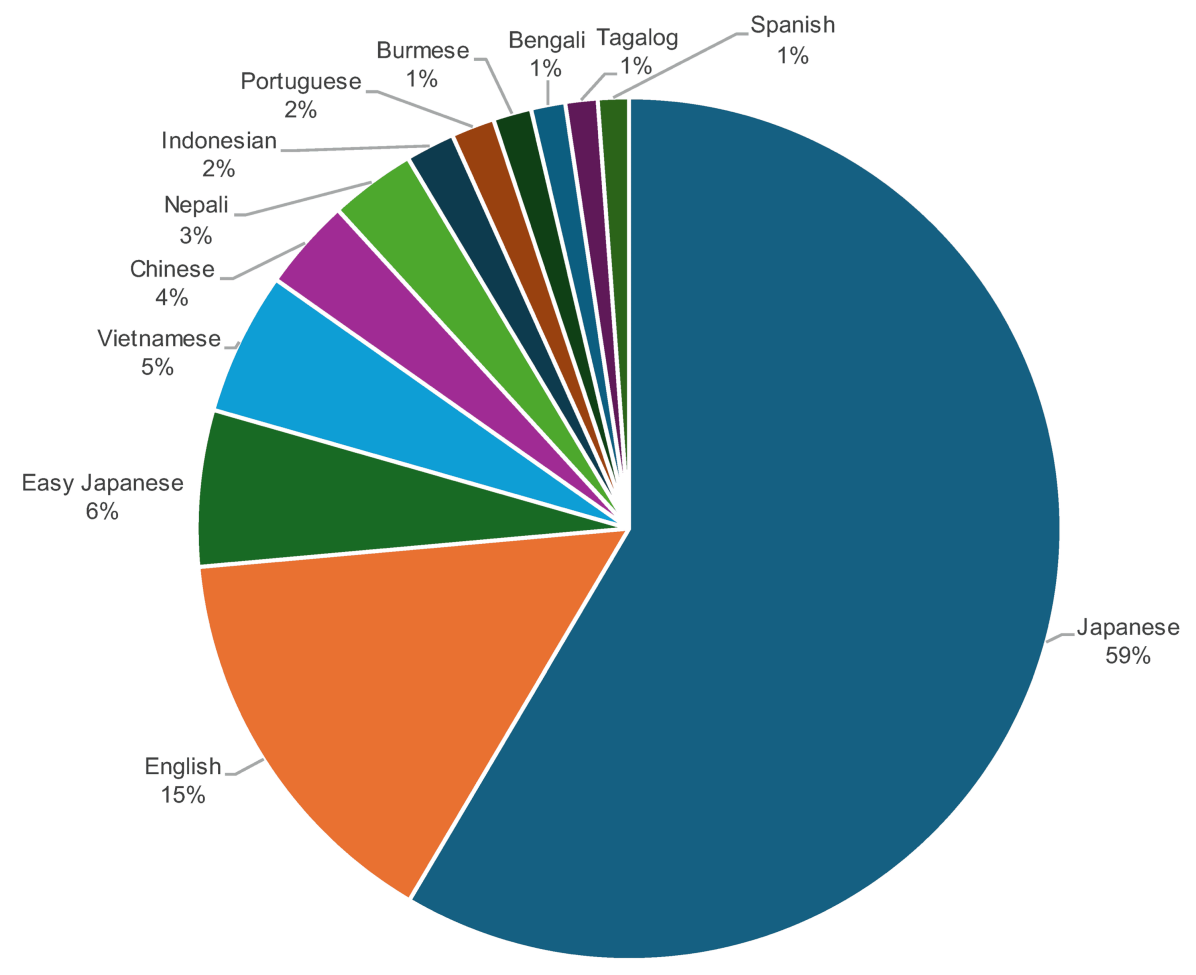
Percentage of page views by language

A language-specific analysis revealed that the majority of users accessed content in Japanese (59%; n = 96,397), followed by English (15%; n = 24,872), Easy Japanese (6%; n = 9,658), Vietnamese (5%; n = 8,834), and Chinese (4%; n = 5,671). Other languages, including Nepali, Indonesian, Portuguese, Burmese, Bengali, Tagalog, and Spanish, represented smaller percentages of total access (Figure 2).

Frequently accessed content areas included immunization schedule, parenting chart, Living and Working Guidebook, child development information, and Maternal and Child Health Handbook (Table 1).

**Table 1:** Number of page views for each material by language

Material	Japanese	English	Easy Japanese	Vietnamese	Chinese	Nepali	Portuguese	Indonesian	Burmese	Bengali	Spanish	Tagalog	Total
Immunization schedule	3688	1103	386	361	213	196	90	149	142	107	88	87	6610
Parenting chart	3307	969	484	473	215	271	72	80	78	68	79	40	6136
Living and Working Guidebook	2497	1515	454	512	318	180	88	207	130	83	63	66	6113
Child development information	2452	896	460	193	156	150	99	102	174	49	51	32	4814
Maternal and Child Health Handbook	2682	657	213	244	195	48	208	101	84	36	23	35	4526
Support series for mothers and babies - information on pregnancy and childbirth	2595	614	331	240	169	124	59	113	67	47	38	53	4450
Communication tools for newborn and baby visitors	4073	No presentation of materials in foreign languages or in Easy Japanese because they are designed for supporters of foreign-origin parents.											4073
For a healthy pregnancy and delivery (leaflet)	1758	421	262	229	120	62	51	83	77	39	32	46	3180
Parent and child health check (medical questionnaire)	2848	No presentation of materials in foreign languages or in Easy Japanese because they are designed for supporters of foreign-origin parents.											2848
Immunization and child health - 2022 edition	2682	No presentation of materials in foreign languages or in Easy Japanese because they are designed for supporters of foreign-origin parents.											2682
Pointing board for postpartum checkups/visits	970	171	308	67	34	20	13	33	18	33	6	12	1685
Prenatal and postnatal confirmation sheet	1499	No presentation of materials in foreign languages or in Easy Japanese because they are designed for supporters of foreign-origin parents.											1499
Pointing board useful during childbirth	625	152	212	80	28	18	4	32	26	16	11	6	1210
Multilingual medical questionnaire	1144	No presentation of materials in foreign languages or in Easy Japanese because they are designed for supporters of foreign-origin parents.											1144
Birth plan	378	177	80	79	30	14	31	46	29	14	15	13	906
Health handbook for mothers/fathers from different cultural backgrounds and Japanese health professionals	217	No presentation of materials in foreign languages or in Easy Japanese because they are designed for supporters of foreign-origin parents.											217

Language-specific trends indicated a preference for the immunization schedule among Bengali, Spanish, and Tagalog users; the parenting chart among Easy Japanese and Nepali users; and the Living and Working Guidebook among English, Chinese, Vietnamese, and Indonesian users. Portuguese-speaking users had the highest engagement with the Maternal and Child Health Handbook. Among Japanese-language users, a notable volume of access was directed toward the section on communication tools for newborn and baby visitors, suggesting a strong demand for practical tools to support caregiver communication.

Access was recorded from 1,149 unique locations. After excluding 22 entries with unidentified locations, 1,127 remained. Of these, 369 originated from 65 foreign countries. The remaining 757 municipalities spanned all 47 prefectures in Japan, accounting for 44.06% of the nation’s 1,718 municipalities.

The analysis identified the 20 municipalities with the highest frequency of website access, together with their national rankings in terms of foreign resident populations (out of 1,718 municipalities), as reported in official statistics [17].

These municipalities were Shinjuku City (ranked 8th nationwide in the number of registered foreign residents), Osaka City (1st), Katsushika City (20th), Fukuoka City (7th), Yokohama City (2nd), Nagoya City (3rd), Minato City (24th), Saitama City (16th), Kobe City (5th), Mito City (169th), Chiyoda City (175th), Shibuya City (53rd), Sapporo City (36th), Sakai City (37th), Nakano City (23rd), Hiroshima City (27th), Chiba City (13th), Fujieda City (298th), Kyoto City (4th), and Kawasaki City (6th).

User evaluation

A total of 39 responses were obtained from the user satisfaction survey, comprising all individuals who voluntarily participated during the data collection period (April 2020-March 2025), with no inclusion or exclusion criteria applied. The majority were from English (n = 17) and Nepali (n = 10) speakers. All respondents reported that the website text was “easy to read.” Regarding content comprehension, 35 respondents rated it as “easy to understand,” three as “somewhat easy,” and one as “somewhat difficult.” Regarding usability, 31 respondents rated the website as “easy to use,” seven as “somewhat easy,” and one as “difficult.”

Qualitative feedback was obtained through optional open-ended responses in the user satisfaction survey. Six respondents provided comments, including statements such as “everything is good” (n = 1), “rich in content” (n = 1), “easy to understand” (n = 1), and “would like the content to be further expanded” (n = 1). Based on this feedback, the website has gradually expanded its content over time.

Analysis of inquiries

A total of 33 inquiries were received over the five-year period: 17 in 2020, six in 2021, four in 2022, five in 2023, and one in 2024. Each inquiry was followed up with an individual response to address the user’s request or concern.

Of these, 23 were submitted by Japanese individuals supporting foreign residents, including public health center staff, members of support organizations, and other affiliated personnel. The content primarily concerned requests for permission to feature the website on organizational websites or social media (n = 8), requests for website flyers (n = 6), and requests to publish information in magazines (n = 3). Additional inquiries included a request for permission to use materials (n = 1), a request for the Japanese version of the parent and child health check questionnaire (n = 1), requests for materials in Khmer (n = 1) and Urdu (n = 1), suggestions regarding workshops for children with disabilities in heritage languages (n = 1), and a volunteer application (n = 1).

Ten inquiries were presumed to have been submitted by foreign parents. These inquiries addressed a wide range of issues, including requests for school meal support at elementary schools (n = 2), questions about health insurance coverage for children after birth (n = 1), inquiries regarding safe vitamins or supplements during pregnancy (n = 1), concerns about how to respond to a crying infant (n = 1), and expressions of anxiety related to parenting (n = 1). Other inquiries included requests for materials in native languages (n = 1), questions about child-rearing practices (n = 1), comparisons of immunization schedules between home countries and Japan (n = 1), and accounts of mental and physical fatigue associated with court proceedings involving the detention of a spouse (n = 1).

## Discussion

The results of this five-year evaluation highlight the utility and impact of a multilingual digital platform in addressing information access gaps for foreign-origin families in Japan. The following discussion integrates insights from website usage patterns, user evaluations, and an inquiry analysis.

Website access and geographic distribution

The website’s extensive reach, spanning 757 municipalities across all 47 prefectures, underscores its national relevance and increasing visibility. Notably, municipalities with the largest foreign resident populations also recorded the highest levels of website access, suggesting that the platform is effectively utilized in areas with substantial multicultural communities. Although municipality-level data disaggregated by both nationality and age group are not publicly available, population pyramids for foreign residents in Japan indicate that a large proportion are in their late teens to 30s, the age range most commonly associated with pregnancy, childbirth, and child-rearing. Therefore, it is reasonable to assume that municipalities with larger foreign resident populations also include relatively more individuals in the child-rearing generation. Nevertheless, this interpretation should be made with caution, and future studies incorporating age- and nationality-specific data would enable a more precise examination of this relationship. Despite these limitations, only 44.06% of Japan’s municipalities recorded access to the website, highlighting opportunities for strategic expansion through targeted outreach, particularly in rural or underserved areas. Collaborations with local governments and non-government organizations (NGOs) could further enhance the platform’s reach.

Language accessibility and content relevance

The usage data and inquiry content collectively reflect language-specific needs. The high engagement with Easy Japanese and English suggests that these languages are effectively reaching a diverse user base, including individuals with limited Japanese proficiency. By contrast, the lower access rates observed for certain minority languages may indicate barriers such as limited awareness of the website or broader systemic challenges, including digital exclusion. Digital exclusion refers to restricted access to online resources due to difficulties in obtaining internet connectivity or digital devices. Enhancing awareness among these language communities may require tailored promotional strategies and collaboration with community-based organizations.

Content engagement and relevance

User interaction data showed that practical and actionable content, such as immunization schedules, parenting tips, and resources on living and working in Japan, was the most frequently accessed. These results are consistent with previous studies indicating that migrant families seek trustworthy and contextually relevant health information to support everyday decisions in unfamiliar healthcare systems [18,19].

Opportunities for outreach

The website was accessed from 757 municipalities across all 47 prefectures, indicating a broad national reach and relevance. However, this represents only 44.06% of all municipalities in Japan, suggesting that gaps in information access remain, particularly in underserved or rural areas. While digital platforms such as this website can help overcome geographic barriers by providing accessible information nationwide, additional efforts are needed to ensure equitable reach. Potential strategies include distributing printed materials, organizing informational sessions through local governments and public health centers, collaborating with foreign communities, support organizations, and NGOs to share information, and promoting the platform through social and local media. Integrating digital access with these targeted outreach activities may strengthen information dissemination and help bridge both geographic and social gaps.

User feedback and implications

Findings from the user satisfaction survey suggest that the platform has largely achieved its original goal of providing clear, understandable, and usable health information for foreign residents. The consistently high ratings for readability, comprehension, and usability indicate that its design and language approach are appropriate for diverse user groups. Qualitative feedback highlighted the value of bilingual content in medical contexts and emphasized the need for further expansion of language coverage and content diversity.

At the same time, this feedback identified areas for improvement after five years of implementation. Requests for more comprehensive information and broader language coverage suggest that some user groups may still encounter barriers to fully utilizing the platform. These findings underscore the importance of regularly updating and expanding content in response to evolving user needs, extending language coverage, and collaborating with healthcare providers and local governments to integrate the platform into routine health communication practices.

Inquiry analysis and stakeholder engagement

The analysis of inquiries provided valuable qualitative insights into the website’s role for both end users and intermediary supporters. The predominance of inquiries from Japanese professionals regarding the use of materials, media requests, and event-related support illustrates that the platform serves not only as a user resource but also as a practical tool for public health, education, and social work practitioners. This dual functionality reflects findings in the literature emphasizing the importance of accessible digital tools to enhance service coordination in multicultural health environments [11].

Inquiries from presumed foreign users, although fewer in number, revealed practical concerns related to everyday parenting, navigating health insurance, and responding to emotional or legal stress. These topics point to unmet needs and suggest directions for future content expansion, including mental health, legal rights, and culturally sensitive parenting resources. Similar patterns of psychosocial stress and information gaps among migrant families were previously reported [4,11]. The nature of these inquiries also emphasizes the value of maintaining and promoting the multilingual inquiry function, which aligns with global recommendations for inclusive, linguistically responsive health communication systems [20].

Implications for multilingual health communication

The trends observed herein validate the significance of multilingual digital platforms in supporting maternal and child health equity. The alignment between frequently accessed content (e.g., immunization schedules and parenting charts) and common inquiry themes highlights the importance of practical, life-oriented resources. Previous studies showed that linguistically appropriate materials may facilitate the timely utilization of services and reduce parental anxiety among immigrant families [5,8].

Bilingual content that is culturally and linguistically appropriate not only supports foreign families directly but also enhances the capacity of local service providers. This result is consistent with emerging literature on the role of digital platforms in enabling equitable access to care, particularly when information is co-designed or curated with cultural sensitivity [12,21].

Future recommendations

To further build on the current achievements, it is recommended that the platform continue expanding its linguistic coverage, develop interactive and scenario-based tools such as chatbots and videos, and conduct outreach activities tailored to minority language speakers. Beyond improving access to information, future initiatives should clarify and strengthen the process by which users receive, understand, and apply information in their daily lives. For example, providing practical guides or step-by-step instructions on topics such as infant health checkups or how to access maternal and child health services could support users in translating information into action. In addition, incorporating user feedback continuously through periodic surveys and inquiries will be essential to adapt to the evolving needs of communities and to ensure that information is not merely available but also actionable. Furthermore, future iterations of the platform should clearly inform users about the potential research use of anonymized analytics data and, where feasible, provide an opt-out mechanism. This approach will strengthen the project’s ethical foundation and uphold users’ autonomy and privacy. Taken together, these efforts will enhance the platform’s role as both an information hub and a practical tool for foreign residents and service providers.

In summary, the platform’s effectiveness is demonstrated not only through quantitative web metrics but also via the qualitative nature of user engagement. By combining multilingual access with contextualized health information, the site contributes meaningfully to reducing health disparities and fostering inclusive public health infrastructure in Japan.

Policy and practice implications

The findings of this study provide important actionable insights for policymakers, healthcare providers, and local governments. For policymakers, the high demand for multilingual, practical health information underscores the need to integrate language accessibility into strategies for improving foreign residents’ access to health services. Healthcare providers working in public health centers, clinics, and hospitals can promote culturally responsive care by incorporating multilingual materials into guidance and support related to pregnancy, childbirth, and parenting. Local governments, even those without in-house translation capacity, can enhance information access by disseminating multilingual materials through the platform. Collectively, these efforts are expected to reduce information gaps, facilitate timely access to essential services, and ultimately advance health equity.

Study limitations and strengths

This study has several limitations that must be considered. First, the descriptive design limits the ability to draw causal inferences between website use and specific health outcomes. While Google Analytics provided valuable insights into usage patterns, it did not extract key demographic data such as age, nationality, residency status, education level, or reasons for accessing the website. These omissions restrict a full understanding of users’ characteristics and needs.

To partially address this gap, we conducted a multilingual user satisfaction survey. Although the sample size was relatively small (n = 39) and may not fully reflect overall user satisfaction, participants were primarily English and Nepali speakers, and the findings may reasonably represent the needs and preferences of these groups. Therefore, caution is warranted when generalizing the results to other language communities. In addition, the study relied heavily on quantitative self-reported data, which may not fully capture the complexity of user experiences or the platform’s impact on behavior. Future research should aim to include a larger and more diverse participant pool and incorporate qualitative methods to provide a richer and more nuanced understanding of user experiences across languages. Moreover, although the study methods are described in sufficient detail to ensure transparency, reproducibility depends on access to the website’s user data, which may require permission from the website owners. Access to such data is subject to the platform’s policies and ethical considerations. Where feasible, anonymized or aggregated data could be made available upon request to support reproducibility.

Despite these limitations, the study has several strengths. It analyzed a five-year dataset covering all 47 prefectures and 757 municipalities, reflecting a broad geographic reach. The combination of analytics, survey data, and qualitative inquiry analysis helped cross-validate the results using different data sources. The multilingual approach, including Easy Japanese, addressed accessibility across diverse user groups. These strengths support the study’s contribution to the field of multilingual health communication.

## Conclusions

This study demonstrated that multilingual and culturally appropriate digital platforms can help reduce barriers to maternal and child health information for foreign-origin families in Japan. By providing content in 11 languages, including Easy Japanese, the evaluated website effectively supported both families and healthcare professionals, particularly through practical resources such as immunization schedules and parenting guides. However, limited access in rural areas and among minority language groups highlights the need for targeted promotion and content expansion. Sustained development, co-creation with communities, and integration into broader health service systems will be essential to strengthening health equity in Japan’s increasingly multicultural society.
